# Molecular Pharmacology of Human Metabolism Diseases

**DOI:** 10.3390/ijms26178701

**Published:** 2025-09-06

**Authors:** Łukasz Bułdak

**Affiliations:** Department of Internal Medicine and Clinical Pharmacology, Medical University of Silesia, Medykow 18, 40-752 Katowice, Poland; lbuldak@sum.edu.pl

Despite significant advances in therapeutic lifestyle changes and novel pharmacotherapies, the healthcare burden of metabolic diseases (particularly obesity and its associated complications, such as type 2 diabetes, hyperlipidemia, and fatty liver disease) remains substantial. Recently, several effective pharmacological therapies, particularly GLP-1 receptor agonists, have been introduced for obesity treatment. However, there is a need for a greater focus on other metabolically important organs, especially striated muscles, which can dissipate excess energy, improve insulin sensitivity, and enhance patients’ well-being and overall quality of life. Some of the pertinent issues have been addressed in the current Special Issue of *International Journal of Molecular Sciences*, entitled “Molecular Pharmacology of Human Metabolism Diseases.” Here, we have gathered a selection of studies that focus on weight management and its impact on obesity-related metabolic complications, as well as research on improving muscle function.

In the first study, the authors presented an interesting clinical trial on a 3-month treatment with liraglutide and its effects on markers of liver steatosis in obese subjects without diabetes (contribution 1). Liraglutide acts by activating the GLP-1 receptor, which is involved in enhancing insulin secretion and improving glucose metabolism, a key factor in reducing liver fat content. The drug’s dual action on insulin sensitivity and liver steatosis is supposed to be a key element in its effectiveness in treating Metabolic Dysfunction-Associated Steatotic Liver Disease (MASLD). While weight loss plays an essential role in metabolic diseases, it is not completely clear how liraglutide’s pharmacological actions directly affect hepatic function. During the course of the study, a significant reduction in body weight was observed, followed by improvements in liver steatosis, assessed by ultrasound-based techniques (Fibroscan) and clinically relevant, easily accessible serum markers such as those used to calculate the Hepatic Steatosis Index (HIS). Another notable finding was the variability in the performance of insulin resistance algorithms. In the case of MASLD, those based on triglyceride levels seem to be more appropriate. In summary, the results of the study underline the importance of effective pharmacotherapy for obesity that, to some extent, can reverse metabolic complications, i.e., MASLD.

The following study by Kramar et al. (contribution 2) focused on oxidative stress induced in Fao rat hepatocarcinoma cells during in vitro exposure to olanzapine or aripiprazole, which are both atypical antipsychotics. Previous research indicates that prolonged therapy with these drugs may increase cardiovascular risk, partly due to appetite-stimulating effects and resultant weight gain associated with olanzapine. Olanzapine tends to increase mitochondrial reactive oxygen species (ROS), which may exacerbate mitochondrial dysfunction and oxidative stress in liver cells. In contrast, aripiprazole-treated cells exhibit resistance to oxidative stress. Further experiments showed divergent responses to oxidative stimulus in the presence of olanzapine and aripiprazole, which shows potential link between oxidative stress pathways and those regulated by mitogen-activated protein kinases (MAPKs) and signal transducer and activator of transcription 3 (STAT3), which are essential in determining cell survival. As a result, compared to olanzapine, the overall activation of pro- and anti-inflammatory pathways in Fao cells exposed to aripiprazole was blunted, showing desensitization to stress signaling. This study underscores the importance of evaluating long-term drug effects on mitochondrial function, as oxidative stress can contribute to carcinogenesis and inflammation.

Maintaining muscle mass is essential during therapeutic lifestyle changes that include weight management. Many patients are unable or unwilling to perform physical exercises during weight reduction and subsequently suffer from muscle mass decline. Current efforts focus on pharmacological activation of striated muscles to improve metabolism, increase muscle mass, and enhance muscle strength. In this Special Issue, Cook et al. (contribution 3) explored the impact of mTORC activation in enhancing C2C12 myotube metabolism and the potential involvement of branched-chain amino acids in this process. Another study in this collection, focusing on the function and performance of striated muscles, was conducted in vivo using rats with induced pulmonary hypertension (PH) (contribution 4). Authors demonstrated that activation of the mitochondrial deacetylase SIRT3 by Honokiol, in conjunction with supplementation of its co-factor, nicotinamide adenine dinucleotide (NAD), improved exercise capacity. This was attributed to reduced proteolysis, mitigated muscle atrophy, and increased mitochondrial protein levels. These two studies provide promising data on potential pharmacological approaches to improving striated muscle performance, which could lead to improved clinical outcomes. Further studies in this area are warranted.

The final article in the collection is a systematic review with a subsequent meta-analysis focusing on the use of agomelatine, a novel antidepressant, in patients with type 2 diabetes (contribution 5). Agomelatine is unique in being both a melatonin receptor agonist and a serotonin 5-HT2C receptor antagonist, which might contribute to improvement in glycemic control and reduction in depressive symptoms. Mood disorders are common in patients diagnosed with diabetes, so it is not unusual for diabetic patients to also receive treatment for depression or anxiety. Choosing a drug with minimal metabolic impact is crucial. The authors of the meta-analysis found that, compared to other serotonin reuptake inhibitors (SSRIs), agomelatine was at least equally effective in treating psychiatric symptoms but had a significantly better impact on metabolic glucose control. The authors concluded that, based on their findings, agomelatine might be a suitable treatment option for diabetics with depression or anxiety.

A common theme across the studies in this Special Issue is the critical role of oxidative stress, inflammation, and lipid metabolism in metabolic diseases. Liraglutide and agomelatine show promise in improving metabolic function and insulin resistance in clinical settings. Preclinical studies, on the other hand, underline the importance of targeting oxidative stress and mitochondrial dysfunction using novel approaches. In summary, these studies provide valuable insights into therapies targeting striated muscles and the liver to achieve metabolic equilibrium, emphasizing the need for personalized, targeted therapies, particularly in patients with comorbidities.

## Data Availability

Not applicable. No new data obtained.

